# Evaluating health system barriers to sexual and reproductive health service delivery during the COVID-19 pandemic in China: a mixed-methods study

**DOI:** 10.1186/s12978-025-02093-z

**Published:** 2025-09-22

**Authors:** Hanxiyue Zhang, Hao Wang, Xizhuo Xie, Angela Y. Xiao, Moazzam Ali, Caron Kim, Grace H. M. Kapustianyk, Kun Tang

**Affiliations:** 1 Vanke School of Public Health, Tsinghua University, Beijing, China; 2 Department of Epidemiology, Faculty of Medicine, Prince of Songkla University, Hat Yai, Songkhla, Thailand; 3 Department of Sexual and Reproductive Health and Research, WHO, Geneve, Switzerland

**Keywords:** COVID-19 pandemic, Sexual and reproductive health, Health system, Family planning, China

## Abstract

**Background:**

The disruption of sexual and reproductive health (SRH) services emerged as a key issue during the early COVID-19 outbreak. We aimed to assess the availability of SRH services in China during the COVID-19 pandemic.

**Methods:**

The study is part of a larger cross-country study on the impact of COVID-19 pandemic on health system. A cross-sectional panel survey design with two data collection points was used to capture changes in SRH service availability as a result of the COVID-19 pandemic. We developed a questionnaire to assess the readiness and resilience of China’s health system. We conducted 109 in-depth interviews with healthcare providers, clients, and their partners in seven purposively selected health facilities in Wuhan, Beijing, and Changsha between November 2020 and December 2022. An adapted grounded theory and thematic analysis approach were applied to analyze the interview data. Direct quotes and findings from the coding and categorization process were used to develop the results.

**Results:**

The quantitative data showed that family planning, including contraception, and safe abortion services were completely or partially disrupted by the COVID-19 pandemic. The effects were greater at baseline. The disruption of services can be attributed to both supply-side and demand-side factors. Facilities responded to the pandemic’s adverse impact on essential health services in multiple ways, most commonly through telemedicine, task shifting/role delegation, and triaging, to prioritize resource allocation. Three major themes emerged from the qualitative data: (1) barriers to SRH service delivery, (2) barriers to access and utilization of SRH services, and (3) resilient innovations in response to COVID-19. Service providers experienced barriers in providing SRH services to women during the pandemic. Women also faced numerous barriers and challenges to accessing SRH services, including lockdowns and mobility restrictions, lack of access to information, and limited access to SRH products. Most participants expressed support for the further expansion of telehealth strategies to improve access to SRH resources.

**Conclusions:**

The COVID-19 pandemic negatively impacted SRH service provision and utilization in China. Our findings highlight the challenges of SRH service delivery during the COVID-19 pandemic and can be used to guide policy development and enhance service provision in alignment with societal needs. These findings underscore the importance of strengthening health systems by supporting telehealth expansion, tailoring provider–client communication, addressing the gender-specific needs of female providers, and leveraging community engagement to sustain essential SRH services during future crises.

**Supplementary Information:**

The online version contains supplementary material available at 10.1186/s12978-025-02093-z.

## Plain Language Summary

Sexual and reproductive health (SRH) services are vital for women, and disruptions in services can lead to unwanted pregnancies, sexually transmitted diseases, and increased health risks for mothers and their newborns. However, during the COVID-19 pandemic, many essential health services, including SRH services, were suspended. Access to SRH products and services was limited due to public health constraints, which may have negatively impacted women's SRH. The effects of COVID-19 restrictions on women’s access to SRH services in China have not been adequately explored. Moreover, little is known about the impact of the pandemic on the provision and utilization of SRH services. This study aimed to evaluate health system barriers to SRH service delivery in China during the COVID-19 pandemic. We used a questionnaire to explore the readiness of SRH services during the pandemic. In addition, we conducted qualitative interviews with women seeking SRH services, their partners, as well as healthcare providers to understand their perceptions of how the pandemic affected care-seeking behavior and service provision. This paper provides an in-depth analysis of the barriers women faced in accessing SRH services during the pandemic, as well as resilience and coping strategies, particularly in the areas of community engagement and digital counseling. We found that improved communication from healthcare providers, support for female health workers, and expanded use of telehealth are key strategies that can help maintain access to SRH services during emergencies. In addition, community involvement, which helps people stay informed and connected to care, should be strengthened in future responses. The study findings will help policymakers and health managers to develop and strengthen policies and services that are more responsive to community needs.

**Supplementary Information:**

The online version contains supplementary material available at 10.1186/s12978-025-02093-z.

## Background

Coronavirus disease 2019 (COVID-19) emerged in December 2019 in Wuhan, China and rapidly spread across the country within two weeks [1]. By the end of January 2020, the Chinese government had initiated emergency responses from the national to the local levels. A series of measures were implemented to alleviate the impact of the pandemic, including the establishment of large temporary hospitals, city lockdowns, patient referral and isolation measures, and quarantine for high-risk individuals. A huge number of resources, especially medical resources, were put into China’s COVID-19 response.

China's health system faced dual pressures during the pandemic. On one hand, the health system had to maintain its original functions to ensure the provision of essential health services. On the other hand, the health system needed to adapt to the new challenges and demands brought about by COVID-19, which required substantial changes to the delivery of health services. Together, this posed significant political, economic, and organizational-level challenges. The health system faced formidable challenges in the early stages of the pandemic, including a shortage of medical supplies and equipment, disruptions in transportation, and contractions of the virus among healthcare personnel [2].

Sexual and reproductive health (SRH) and rights constitute a longstanding public health priority, and its importance was especially evident during the COVID-19 pandemic. The pandemic revealed a range of clinical- and health system-level challenges, including the redeployment or shortage of healthcare personnel in SRH services, increased demand for safe abortion care, heightened risks of gender-based violence and domestic abuse under quarantine and mobility restrictions, and the rise of stigma and discrimination associated with COVID-19 related conditions [3]. These issues severely impeded the health system's ability to deliver routine SRH services for women during this period, thereby impacting the overall health and well-being of women [4]. Major disruptions to the provision of SRH services during the pandemic exposed women to significant health risks, including unwanted pregnancies, unsafe abortions, gender-based violence (GBV), and intimate partner violence (IPV), among others [5].

Several studies have examined the impact of the COVID-19 pandemic on SRH [6–9]. The United Nations Population Fund (UNFPA) estimated that the pandemic disrupted contraceptive use for about 12 million women worldwide, which resulted in nearly 1.4 million unintended pregnancies across 115 low- and middle-income countries in 2020 [10]. At the same time, disruptions in the supply chain led to a shortage of contraceptives, an issue that was more pronounced in developing countries [11]. In addition, a study conducted in China showed that the proportion of women seeking abortion services due to social factors increased significantly as a result of the pandemic [12].

The aforementioned studies primarily focus on patients’ physiological health using clinical and laboratory data, with limited examination of the broader health system. They are largely descriptive in nature and lack qualitative inquiry into the functioning and evolution of China’s health system during the pandemic. Moreover, diverse perspectives—such as those of women seeking care and their partners—remain underexplored. The main aim of our research is thus to investigate the provision of SRH services by local health facilities in China during the pandemic. Our research findings, which investigate and offer recommendations to relevant stakeholders, may help enhance health system resilience, optimize functionality, and improve health system response to future pandemics or other challenges.

## Methods

### Study design

This study adopted a mixed-methods approach combined with a panel design, incorporating both quantitative and qualitative data collection at two time points, baseline and endline. The baseline survey collected data between January 2021 and June 2021, focusing primarily on the outbreak phase of the pandemic, its impact on the health system, and the changes implemented. The endline survey collected data between June 2022 and December 2022, with a primary emphasis on the new challenges encountered by the health system two years after the initial outbreak and the methods employed to address these challenges. The World Health Organization (WHO) Service Availability and Readiness Assessment (SARA) guide and WHO Health Facility Readiness Checklist were used to conduct assessment of health system infrastructure and service provision [13, 14]. In-depth interviews were conducted to understand the participants’ perspectives of SRH service availability and readiness in COVID-19-affected areas.

### Study setting and participants

This study is part of a broader research project on health systems analysis and evaluations of the barriers to availability, utilization, and readiness of SRH services in COVID-19 affected areas, initiated by the WHO [15]. The study was conducted in Beijing, Wuhan, Hubei Province, and Changsha, Hunan Province. To ensure variation in response, we selected seven health facilities across the three cities to serve as the focal points, taking into consideration hospital capacity, administrative rank, the local city’s level of urbanization, and willingness to participate in the study to capture diverse perspectives across different tiers of health facilities within the health system and various geographic settings. The selection of study sites is shown in Table S3 (Appendix). In Beijing, the selected facilities included a tertiary-level hospital and a secondary-level hospital located in urban areas, as well as a community health center in a suburban area. In Wuhan, we selected a secondary-level hospital in an urban area and a community health center in a suburban setting. In the endline survey, the two health facilities in Wuhan were not able to participate in the study as planned. To mitigate this, we selected Hunan, (a province adjacent to Hubei province) to conduct the survey. Because China was experiencing various new waves of the pandemic in 2022, despite being two different provinces, the situations and associated health system responses were similar. Thus, we included a tertiary-level hospital in an urban area of Changsha, Hunan Province and a community health center in the same city. This sampling strategy allowed for a diverse representation of institutional characteristics and local contexts within the broader Chinese health system.

The study population included women seeking SRH services and their partners as well as healthcare providers (HCPs) in obstetrics (OB), gynecology (GYN), and/or family planning (FP) departments. For the quantitative component, one to two healthcare providers were selected from each health facility to assist with the facility assessment. These individuals were purposively chosen based on their knowledge of SRH services at the facility, with selection criteria including seniority, professional role, and familiarity with SRH service delivery. The providers were eligible for inclusion if they (1) were knowledgeable about the SRH services provided in the facility and (2) provided written informed consent to participate in the survey and in-depth interview.

The sample size for the qualitative in-depth interviews was guided by the principle of data saturation, with emphasis on whether each healthcare facility had provided sufficiently rich information. As previously described, we employed data triangulation across multiple data sources—including healthcare providers, women, and their partners—to enhance the study’s internal validity. In the hierarchy of priorities, direct perspectives from women accessing SRH services were central, followed by insights from healthcare providers, with partners’ viewpoints serving as complementary contributions; this prioritization informed the allocation of sample sizes across participant groups.

Saturation was operationally defined as the point at which three independent interviewers collectively agreed that no new themes emerged from the last three consecutive interviews for a given facility and that all research questions had been comprehensively addressed. This criterion ensured that diverse stakeholder perspectives—prioritizing women’s experiences—were sufficiently explored to achieve theoretical depth, with provider and partner insights enriching the interpretive framework through cross-source validation enabled by data triangulation.

The inclusion criteria of the women were: (1) lived in the study sites, (2) of reproductive age (18–49 years), (3) sought or tried to receive reproductive health services from local health facilities during the pandemic, and (4) provided written informed consent to participate in the in-depth interview. Considering that including minors (15–17 years old) would require additional ethical approval and guardian consent, which could affect the feasibility of recruitment and data quality, this group age was excluded from the study. The inclusion of partners was determined by the inclusion of women. That is, the partners of women who had already been interviewed by us were invited to participate in the interviews.

### Data collection

Data were collected between November 2020 and December 2022 through a semi-structured interview guide, which was co-developed with the WHO and included demographic information, knowledge about COVID-19, the problem the women sought care for, care-seeking behavior, and perception of risk of COVID-19 infection. Throughout the data collection process, the interview guide was adjusted based on the different study sites and new findings. Each interview lasted between 40 to 60 min and was recorded. The audio files were anonymized along with other qualitative data. All personally identifiable information of the participants was removed, and each participant was given a unique number to protect privacy. All audio files were professionally transcribed verbatim. The quantitative data were collected using paper-based questionnaires. To ensure the validity and reliability of the data collected, a pilot study was conducted to ensure the validity of the survey questionnaire which was adopted based on various assessment tools. The principal investigator and project team were trained on the study purpose, design, quality assurance, reporting mechanisms, and data collection tools. All forms were routinely checked for completeness, logical errors, and unclear or irrelevant responses. All data were double-entered by two independent data operators to ensure accuracy. The investigator and project team conducted regular quality checks and spot-checks to ensure data quality and adherence to the protocol.

### Ethical approval

Ethical approval for this study was obtained from the ethics review committee (ERC) of Tsinghua University (approval No. 20200040) and ERC of WHO (approval No. CERC. 0027). All participants were informed about the objectives of the study and their rights, and provided written informed consent before the interview.

### Data analysis

We analyzed data using adapted grounded theory and thematic analysis approach. First, two independent researchers read the interview transcripts in detail and agreed on a framework for analysis. Second, the researchers separately performed open coding, summarizing the text in words and phrases. Third, each of the two researchers explored the connections in their codes and performed axial coding to identify core categories and sub-categories. Fourth, the two researchers discussed with each other to reach a consensus on the core categories and performed selective coding to dissolve unfocused categories and codes before finally constructing a coding tree. Fifth, the entire research team then discussed the coding tree to determine the coherence and logic of the codes. Lastly, the team reviewed the transcripts to confirm that the text provided sufficient evidential support for the coding tree and that there were no key themes left out of the codes. All interview transcripts were entered into the Dedoose (version 9.0.107), a qualitative data management and analysis software.

## Results

### Participant characteristics

A total number of 29 HCPs and 80 women and their partners were recruited and interviewed. The numbers of participants in the baseline and endline surveys are shown in Table S2 (Appendix). The participant characteristics are presented in Tables 1 and 2, respectively.

**Table 1:** Demographic characteristics of healthcare providers (*n* = 29)

Subject number	Sex	Age	Professional title	Medical institution	Ethnicity	Years of work
1101	Female	51	Head physician	Grade 3 A hospital	Han	24
1102	Female	48	Head physician	Grade 3 A hospital	Han	24
1103	Female	42	Deputy head physician	Grade 3 A hospital	Han	10
1104	Female	34	Attending physician	Grade 3 A hospital	Han	6
2101	Female	32	Attending physician	Grade 2 A hospital	Han	7
2102	Female	48	Deputy head physician	Grade 2 A hospital	Han	23
2103	Female	44	Deputy head physician	Grade 2 A hospital	Han	20
3101	Female	33	Assistant physician	Community health center	Han	10
3102	Female	42	Deputy head physician	Community health center	Han	20
5101	Female	33	Resident	Community health center	Han	10
5102	Female	43	Attending physician	Community health center	Han	16
4101	Female	54	Head physician	Grade 2 A hospital	Han	29
4102	Female	46	Deputy head physician	Grade 2 A hospital	Han	23
4103	Female	40	Deputy head physician	Grade 2 A hospital	Han	17
1153	Female	37	Attending physician	Grade 3 A hospital	Han	14
1155	Female	33	Attending physician	Grade 3 A hospital	Han	6
1156	Female	33	Attending physician	Grade 3 A hospital	Han	3
1157	Female	48	Nurse	Grade 3 A hospital	Han	25
7151	Female	43	Attending physician	Community health center	Han	25
7152	Female	32	Nurse	Community health center	Han	10
7154	Female	36	Nurse	Community health center	Han	14
6157	Female	54	Head physician	Grade 3 A hospital	Han	32
6158	Female	30	Attending physician	Grade 3 A hospital	Tujia	5
6163	Female	55	Deputy head physician	Grade 3 A hospital	Han	30
6172	Female	52	Head nurse	Grade 3 A hospital	Han	33
2163	Female	31	Attending physician	Grade 2 A hospital	Han	9
2165	Female	35	Nurse	Grade 2 A hospital	Han	15
2166	Female	31	Attending physician	Grade 2 A hospital	Han	8
3152	Female	43	Deputy head physician	Community health center	Han	21

**Table 2:** Demographic characteristics of clients and partners (*n* = 80)

Subject number	Sex	Age	Marital status	Education level	Ethnicity
Clients					
1305	Female	33	Married	Graduate school	Han
1306	Female	39	Married	University/College	Han
1307	Female	40	Married	University/College	Han
1308	Female	36	Married	Primary/Elementary 1	Han
2204	Female	37	Married	High school-Vocational school/Secondary	Han
2205	Female	29	Married	University/College	Han
2206	Female	32	Married	University/College	Han
2307	Female	30	Married	Graduate school	Manchu
2308	Female	37	Married	University/College	Han
2309	Female	38	Married	University/College	Han
2310	Female	29	Married	University/College	Han
2311	Female	28	Married	University/College	Han
2312	Female	31	Married	University/College	Han
3203	Female	29	Married	University/College	Han
3204	Female	29	Divorced	University/College	Han
3205	Female	29	Married	University/College	Han
3306	Female	42	Married	High school-Vocational school/Secondary	Han
3307	Female	34	Married	High school-Vocational school/Secondary	Han
3308	Female	25	Married	Primary/Elementary 1	Han
3309	Female	42	Married	University/College	Han
3310	Female	43	Married	High school-Vocational school/Secondary	Han
3311	Female	29	Married	University/College	Han
5203	Female	35	Married	Primary/Elementary 1	Han
5204	Female	41	Divorced	University/College	Han
4204	Female	30	Married	University/College	Han
4205	Female	32	Married	University/College	Han
4206	Female	29	Married	High school-Vocational school/Secondary	Han
4307	Female	41	Married	Secondary school/Elementary 2	Tujia
4308	Female	43	Married	University/College	Han
4309	Female	43	Married	University/College	Han
4310	Female	41	Married	University/College	Han
1354	Female	36	Married	University/College	Han
1358	Female	40	Married	University/College	Han
1368	Female	27	Single	Graduate school	Han
1369	Female	43	Married	Graduate school	Han
7260	Female	38	Married	University/College	Han
7363	Female	42	Married	High school-Vocational school/Secondary	Han
7364	Female	32	Married	University/College	Han
7366	Female	40	Married	University/College	Han
7370	Female	45	Married	University/College	Han
7371	Female	33	Married	University/College	Han
6254	Female	28	Married	University/College	Han
6362	Female	41	Married	University/College	Han
6365	Female	30	Married	University/College	Tujia
6367	Female	41	Married	High school-Vocational school/Secondary	Han
6369	Female	40	Married	University/College	Yao
6371	Female	25	Single	University/College	Han
6376	Female	27	Married	University/College	Han
2251	Female	34	Married	University/College	Han
2355	Female	34	Married	University/College	Hui
2356	Female	29	Married	University/College	Han
2357	Female	43	Married	High school-Vocational school/Secondary	Han
2358	Female	29	Married	University/College	Han
2359	Female	29	Married	University/College	Han
2360	Female	43	Married	University/College	Han
2361	Female	35	Married	University/College	Han
3251	Female	27	Married	University/College	Han
3258	Female	40	Married	University/College	Han
3354	Female	49	Married	High school-Vocational school/Secondary	Han
3356	Female	43	Married	University/College	Han
3359	Female	54	Married	Secondary school/Elementary 2	Han
3373	Female	48	Married	University/College	Han
3374	Female	47	Married	High school-Vocational school/Secondary	Han
Partners					
1418	Male	35	Married	University/College	Han
2421	Male	33	Married	University/College	Han
2422	Male	39	Married	University/College	Han
2423	Male	34	Married	University/College	Han
3420	Male	39	Married	High school-Vocational school/Secondary	Han
3421	Male	25	Married	Secondary school/Elementary 2	Han
3422	Male	35	Married	University/College	Han
4421	Male	33	Married	Graduate school	Han
1459	Male	40	Married	Graduate school	Han
7465	Male	31	Married	University/College	Han
7467	Male	40	Married	University/College	Han
6466	Male	31	Married	University/College	Han
6468	Male	39	Married	High school-Vocational school/Secondary	Miao
6470	Male	39	Married	University/College	Han
2462	Male	35	Married	University/College	Han
3453	Male	50	Married	High school-Vocational school/Secondary	Han
3455	Male	45	Married	University/College	Han

Among the healthcare providers (*n* = 29), all were female, with a mean age of 40.8 years (SD=8.1). There was a range of professional roles: senior physicians (including head physicians and deputy head physicians) accounted for 41.38% (*n* = 12), attending physicians comprised 34.48% (*n* = 10), assistant physicians and residents together represented 6.90% (*n* = 2), and nurses (including head nurses) made up 17.24% (*n* = 5). The providers were employed across primary, secondary, and tertiary medical institutions. Most providers (96.55%) identified as Han ethnicity, and the mean duration of working experience was 16.9 years.

Among the women who participated in the study, the mean age was 36.0 years (SD=6.8). Most were married (93.65%), and the majority had received university/college-level education (69.84%). Nearly all (92.06%) identified as Han ethnicity. Among the partners (*n* = 17), all were male. The mean age was 36.7 years (SD=5.8). All partners were married, and the majority had university/college education (64.71%). The majority (94.12%) also identified as Han ethnicity.

### Descriptive analysis of the health system readiness questionnaire

Descriptive analysis of the quantitative data is presented in Table S1 (Appendix). More health facilities were aware of China's policies to maintain essential SRH services during the COVID-19 pandemic and the financial support provided for this purpose at baseline compared to in the endline survey.

In terms of the maintenance of basic health services, the impact on medical services was found to be greater during the baseline study phase compared to the endline, especially services related to family planning and contraception, safe abortion, and sick child services.

Regarding the reasons for service disruptions and changes, the closure of outpatient disease-specific consultation clinics, decreases in inpatient and outpatient volume, and the dispatch or redeployment of department clinical staff to support COVID-19 emergency response efforts were considered by most health facilities as important factors. In both the baseline and endline surveys, participating health facilities indicated that they used telemedicine to replace in-person consultation and task shifting/role delegation to mitigate disruptions to essential health services.

### Key findings from qualitative data

Three broad themes emerged from the interviews: (1) barriers to SRH service delivery, (2) barriers to access and utilization of SRH services, (3) resilient innovations in response to COVID-19. These themes were then further divided into sub-themes, which are presented below.

1. Barriers to SRH service delivery

### Labor shortages

During the initial months of the pandemic, the Chinese government implemented strict workforce mobility controls, along with comprehensive pandemic prevention, control, and quarantine measures. A large number of nucleic acid testing sites, quarantine sites, and fever clinics were established, which required a significant amount of medical manpower to operate. In response to this, the OB/GYN/FP departments of some health facilities dispatched their staff to support fever clinics. The OB/GYN departments at several primary healthcare facilities dispatched their entire staff workforce to support quarantine sites during the early months of the outbreak, thus halting the provision of most SRH services and leaving only a few staff members on duty for obstetric and gynecological referrals and general emergencies.

There were so many patients with fevers (during the outbreak) that we couldn't care for them all 24 hours a day, so we asked all departments to support the fever clinic, starting from Chinese New Year's Eve (the first day of the Spring Festival holiday). However, we did not stop the general outpatient clinic (for OB/GYN), and we continued to provide abortion and intrauterine device (IUD) insertion/removal services. (4101, Wuhan, HCP)

In addition, some facilities redeployed staff within their maternity units. For example, the restriction of maternal escorts, which allowed for only mothers to be admitted to hospitals, resulted in large gaps in maternal care. To support the provision of OB services during the pandemic, facilities redeployed nursing staff from GYN/FP departments, where outpatient clinics were closed and the number of surgeries dropped sharply, to their OB departments. Redeployed staff were primarily responsible for providing daily care to women during and after birth.

The OB department was a bit short of staff. In the past, after giving birth, the family members could go to the ward to help take care of the mother and the child. However, during the pandemic, there was a period of time when no one was allowed to stay with the postpartum mother, so the medical staff had to shoulder a lot of the work. (1102, Beijing, HCP)

Overall, the COVID-19 pandemic in China negatively impacted the availability of human resources in OB/GYN/FP departments. In some cases, facilities redeployed all staff from the OB/GYN/FP departments elsewhere, leading to the temporary halt of non-essential services, such as non-emergency abortion. Because these facilities did not accept patients seeking such services, these women were thus required to go to other institutions to seek care.

### Reduced routine and acute healthcare service capacity

Due to limited human resources, OB/GYN/FP departments reduced the number of consultations, with some even suspending consultations. Overall, the service capacity of OB/GYN/FP-related institutions decreased as a result of the pandemic. The most significant decreases in service capacity were in GYN surgeries, induced abortions, and FP services such as IUD insertion/removal. In contrast, the reduction in service capacity was more modest in OB surgeries and assisted deliveries due to the higher degree of urgency associated with these services. Health facilities reduced the number of outpatient registrations in response to the reduced capacity of the institutions and the risk of exposure.

Our GYN department was closed in the early stages of the outbreak, and only the general practitioner and nurse from the infusion room were left. (5101, Wuhan, HCP)

### Personal protective equipment shortages

At the beginning of the pandemic, there was a brief period in which OB/GYN/FP departments experienced a relative shortage of personal protective equipment (PPE), including masks, gloves, sanitizer, etc. This was due to a nationwide shortage of protective materials as well as the reallocation of supplies to areas with more severe outbreaks (e.g., Wuhan) and frontline departments (e.g., fever clinics, accident and emergency departments, respiratory departments, etc.) The limited availability of protective materials made it more difficult for providers to sustain the provision of SRH services and increased their risk of exposure during consultations, thus further exacerbating existing human resource constraints.

At the beginning of the outbreak, we didn't have enough PPE, like masks and gowns. For a while, we (OB/GYN department) could get N95 masks, but after a week, we nearly ran out. At that time, we all wore hats, masks, face shields, and general protective clothing, but we couldn't change them every day. (4101, Wuhan, HCP)

Since we didn't have any protective materials, so we had to save as much as we could and reduce the number of outpatient clinics when there weren't so many patients. This could be considered passive manpower constraints. (1103, Beijing, HCP)

### Increased physical and psychological burdens

During the pandemic, the physical and mental health of HCPs was affected by the intensity and heavy burden of their workloads. GYN/FP department medical staff in Wuhan, regardless of whether they were sent out to support the frontlines or stayed behind in their departments to provide daily clinical diagnostic services and treatment, were required to follow strict protective measures, which added to their physical workloads. In addition, the initial outbreak in Wuhan coincided with the Spring Festival (Jan 24, 2020-Feb 2, 2020), a national public holiday in China. Yet, medical staff in OB/FP departments had to extend their working hours, reduce, or even cancel their leave in response to the demand for manpower to support the outbreak. In the endline survey, we retrospectively asked HCPs about their vacation time since the early stage of the pandemic and found that, due to the increased workload caused by the pandemic, instances of extended working hours and canceled vacations occurred frequently in the more than two years following the initial outbreak. Moreover, at the height of the outbreak in Wuhan, HCPs were afraid to go home after work, as they were fearful of increasing their families’ risk of exposure, and instead chose to reside in the hospitals during this period.

In terms of mental health, HCPs in Wuhan mentioned feeling *"scared"* and *"worried"* for a period after the initial outbreak and that they personally knew people who had passed away due to COVID-19. However, as the situation in Wuhan gradually reached a turning point where *"the outbreak was getting under control”,* HCPs felt more *"psychologically relaxed"*.

At the beginning of the pandemic, all of us middle-level cadres (in the department of OB and GYN) had to go to work every day during the Spring Festival holiday without any time off. We were also under a lot of psychological pressure. At that time, we were not sure about the contagiousness of the new virus and the recovery process after getting infected, and we were actually a bit scared. (4101, Wuhan, HCP)

### Unique challenges for female HCPs

Female HCPs experienced unique challenges during the epidemic, primarily in relation to their physiological needs, pregnancy and breastfeeding needs, and work-life balance.

#### Menstrual health and hygiene needs

To meet the physiological needs of female HCPs (menstruation, etc.), hospitals supplied appropriate products, such as adult diapers and menstrual hygiene products. Nevertheless, female HCPs working on the front lines mentioned reducing the frequency in which they changed their menstrual and other physiological products to reduce the number of times they had to change protective gear.

We are trying to save the protective clothing, so we don't eat or go to the toilet. After finishing the eight hours, then we change everything altogether, so it still affects the changing of sanitary napkins. (4101, Wuhan, HCP)

#### Needs during pregnancy and breastfeeding

During the pandemic, female HCPs who were pregnant or breastfeeding received support and care from their colleagues in the hospital units and departments. They would try to get assigned tasks with lower workloads and reduced risk of infection, while other colleagues would take on some of their responsibilities.

Generally, the hospital does not arrange pregnant or breastfeeding colleagues to go to the front lines. For example, our department had two staff members on maternity leave during that time, and while they were breastfeeding, they were not assigned work—other staff went instead. (3102, Beijing, HCP)

#### Work-life imbalances

During the pandemic, female HCPs were required to work long hours at their posts, which prevented them from attending to their familial responsibilities. As previously mentioned, there were also concerns about the potential risk of infection to their families if they returned to their homes after work. To address this, female HCPs tried their best to devote themselves to their job, going as far as residing at the hospitals. For providers with urgent family care needs, the facilities accommodated their schedules as much as possible. At home, the family members of female HCPs also supported them both emotionally and operationally, with partners or relatives taking on more responsibility for family care.

For outposted support work, the facility tries to dispatch people who are not married or who have as little contact as possible with kids, such as those without children. (2101, Beijing, HCP)

Basically, if we go to the quarantine ward, we will be there for about one week to ten days, and then we will return to the hospital. We wouldn't dare go back home. My husband and in-laws took care of things at home. (5101, Wuhan, HCP)

2) Barriers to access and utilization of SRH services

### Lockdowns and mobility restrictions

In order to control the further spread and escalation of the outbreak, the government in Wuhan implemented strict city lockdowns and mobility restrictions in the early phase of the pandemic. During this period, women seeking SRH services faced two main barriers: concerns that visiting hospitals would increase their risk of COVID-19 exposure and city lockdowns that restricted their ability to visit health facilities for care.

(After the city locked down) we had to stay at home and were unable to attend the follow-up visit for IUD insertion. (4307, Wuhan, Woman seeking contraption service)

### Lack of access to information

Lack of access to accurate, real-time information was another barrier preventing women from seeking family planning services. Many patients had to inquire about where to seek abortion services but were unable to find the information they needed through their personal networks or online searches. For example, in family planning services, it was difficult for women to obtain the information they needed.

I had to register online to make an appointment to have an abortion. It was very difficult, because the number of patients was controlled by the hospital, so the number of appointments was very low. (3311, Beijing, women seeking abortion service)

For family planning service information, however, major communication gaps remained. Service providers had limited means of providing accurate and real-time information on family planning services, thus women seeking family planning services were unable to access the necessary information required.

I didn't know which hospitals were providing abortion services at this time. I just had to try them one by one. After visiting the first and second hospital, I found out that it also didn't provide these services, so then I ended up going to a third hospital for consultation. (1305, Beijing, woman seeking abortion service)

### Limited access to SRH products

Women reported experiencing difficulties accessing SRH products (i.e., contraceptives, condoms, pregnancy tests and anti-inflammatory drugs) during the pandemic. Regardless of whether it was due to the subjective opinion that it was *"better not to leave the house"* or lockdown restrictions that prevented them from going out, some patients were unable to obtain the medical goods they needed. In Wuhan, women encountered difficulties accessing medicines or supplies due to urban containment measures. They had to confront the realities of'going to the hospital and being exposed to a high-risk environment'and'not being able to leave the house and pharmacies being closed down'until the outbreak was resolved. These dilemmas were only resolved when the COVID-19 situation improved and control measures were lifted.

I can't buy anti-inflammatory medication for gynecological infections. My medication is prescribed at the hospital and mailed to me. (4309, Wuhan, woman seeking other SRH service)

3) Resilient innovations in response to COVID-19

### Adapting patient-provider communication channels

The women surveyed indicated that the information they needed before seeking services during the pandemic mainly included: 1) the facility’s epidemic situation: the number of patients infected with COVID-19, whether the hospital had set up a fever clinic, and if people infected with COVID-19 were being admitted; 2) patient reception: whether the OB/GYN/FP departments of the hospital were open at regular hours or had resumed operation; and 3) service provision: what services the hospital was providing, whether it could meet their individual needs, etc.

During the pandemic, health facilities released information about their SRH services to the public through both online (e.g., WeChat official accounts) and offline (e.g., community and sub-district offices) channels. To provide the necessary medical service information to women seeking SRH services, some hospitals offered appointment booking and medical service-related information through their WeChat official accounts. In addition, community health centers communicated with women seeking SRH services through family doctors.

We all have the family doctors’ contact information. The director of the Women's Federation is responsible for (the pregnant and postpartum women of) every village. If you have any questions, you should call the doctor in charge of your village, and the doctor will give you guidance. (3102, Beijing, HCP)

The hospital’s WeChat official account provides some information, including information on epidemic prevention and control, when pregnant and postpartum women should go back to the facility for a return visit and when they need to get an examination. (2103, Beijing, HCP)

### Adopting telemedicine and online consultation

During the pandemic, service providers adopted telehealth solutions (phone or video) to deliver care. They contacted pregnant women through personal channels (e.g., WeChat or telephone) or telehealth platforms to provide online consultations.

We had the WeChat of pregnant women, so the way we communicated with them was to send WeChat messages. Sometimes we made calls. For those who didn't have WeChat, we sent them SMS."(3101, Beijing, HCP)

For pregnant women who needed to be quarantined at home due to the pandemic, one-on-one communication and consultation with doctors via online platforms allowed them to receive confirmation on whether they needed emergency care and have their medical needs addressed remotely. Medical consultation services were also accessible through online platforms, which allowed pregnant women unwilling to visit the hospital during the pandemic, especially those who just needed to"*order medical tests*"or"*receive lab results*”, to have most of their medical needs met from home.

In fact, especially for pregnant women, it is necessary to have antenatal examination, but some of them did not dare to come to the hospital for fear of infection. So, there was a lot of online counseling during the pandemic, that is, free online medical consultations by doctors, which I think can still play a certain role. (1103, Beijing, HCP)

### Strengthening community engagement

Community-based organizations took on many roles during the pandemic, including COVID-19 prevention and control, medicine purchasing, and communication and coordination between health facilities and patients. For example, the women reported that during the pandemic, they could purchase the required sexual and reproductive medical goods through local community services. Similarly, HCPs disseminated information to the public about appointments and visits to SRH services through community channels, and community workers and volunteers also had regular telephone follow-ups with pregnant women who had been recorded in their community.

The community called me on a regular basis, and the doctor said that if it was inconvenient to come to the hospital during the pandemic, or if there was an emergency, you could consult online. (3203, Beijing, pregnant woman)

## Discussion

In this study, we examined the experiences of service providers and women seeking SRH services in Wuhan, Beijing and Changsha during the COVID-19 pandemic. We found that during the pandemic, HCPs faced significant demands and challenges while women encountered barriers to accessing SRH services. In response, several innovative strategies were developed, including telemedicine, communication adaptations, and community-based outreach and engagement, which helped build health system resilience in the midst of an evolving epidemic situation.

Based on the quantitative data, we found that essential health services, including family planning, contraception, and safe abortion services, were disrupted due to the COVID-19 pandemic. The main causes for the disruption or change in service utilization were closures of outpatient services and outpatient disease-specific consultation clinics and reductions in outpatient and inpatient volumes. This impact was greater at baseline (January to April 2020), which is consistent with the finding from a survey of 105 countries conducted by the WHO between May and July 2020 on SRH services, which found a disruption in family planning services in 68% of countries, with 9% reporting severe/complete disruption [16]. The reasons for service interruptions found in the WHO survey are also similar to those identified in our study.

During the early stages of the pandemic, HCPs faced significant challenges, including a high risk of exposure and infection, understaffing, lack of PPE, and physiological stress [2, 17, 18], etc. In China, 189 medical teams, consisting of 21,569 health professionals were assembled across China and dispatched to Hubei Province to support the local emergency response during the early stage of the epidemic. The resulting staff shortages in OB/GYN/FP departments may have caused disruptions to the regular provision of SRH services, including essential services such as contraception and abortion services.

The effects of COVID-19 on SRH service delivery, as described by HCPs, included staff shortages due to re-tasking, disruptions to delivery, contraception, and abortion care, PPE shortages, and negative physical and mental health impacts. These findings are consistent with several other studies conducted globally where contraception services and abortion services were found to be partially suspended during the pandemic [19–22]. Moreover, the shortage of PPE increased the risk of exposure for HCPs, further limiting the human resources available [23, 24]. The women in our study also reported challenges they encountered while seeking contraception and abortion services, including city lockdowns and mobility restrictions, the lack of access to information on SRH services, and disrupted access to SRH products. Several studies found that transportation and mobility restrictions limited access to contraceptive services [19, 25]. In addition, the lack of real-time, reliable information, such as SRH facility operating hours and capacity, was one of the barriers faced by women seeking SRH services. As one study in New Zealand showed, young people aged 14–24 years faced barriers to sexual health care during COVID-19 lockdown, including lack of information about service availability [26]. This highlights the need for targeted communication by service providers to minimize the unintended consequences of delaying or missing these services.

A notable finding of our study were the gender-specific needs and challenges faced by female HCPs during the pandemic. This finding has been reported in previous studies. A study conducted in Wuhan found that female HCPs faced considerable dilemmas during the pandemic, arising from the conflicting responsibilities between their roles as HCPs and family caretakers as well as the need to avoid contact with family members to reduce the risk of exposure and infection [27]. Globally, women account for 70% of the health and social care workforce, with higher proportions in the nursing and midwifery professions [28]. It is important to understand the experiences of female HCPs during the COVID-19 pandemic to ensure their health and well-being are better supported during the next health emergency.

We found that telehealth represents perhaps the most significant of adaptations adopted during the pandemic and was rapidly deployed by providers to mitigate the pandemic’s effects on healthcare. Telemedicine was promoted during the outbreak to support continuity of care. A study in Britain showed that among 106 individuals who reported using sexually transmitted infection (STI) testing services, 64.4% accessed services remotely (telephone, video or online) [29]. Telemedicine can improve service accessibility and utilization while minimizing the risk of virus transmission by reducing the need for congregation in confined spaces, such as hospital waiting rooms [30]. Our findings illustrate the advantages of teleconsultation, namely that it provided the women with a means to meeting their SRH needs, especially at the beginning of the pandemic. Service providers supported telemedicine as a complementary approach to traditional service delivery. This is consistent with previous findings, which indicated that the pandemic helped accelerate the use of telemedicine for contraception and abortion services, with clinicians reporting positive experiences and suggesting the further expansion of telemedicine after the pandemic [31, 32]. It should be noted, however, that over-reliance on telemedicine could potentially exacerbate existing inequalities, given that individuals from lower socioeconomic background and those living in rural areas still have relatively limited access to technology resources [33].

Community participation not only facilitates the provision and utilization of health services but is also a key factor in the context of the social determinants of health and health as a human right [34]. Especially in more complex situations such as pandemics, community participation can help support overburdened health systems [35, 36]. Opportunities to engage communities in pandemic response efforts include involving members in intervention design and planning, community entry and trust building, social and behavioral change communication, and so on. Consistent with previous findings [37, 38], our study highlighted the positive role that community engagement plays in pandemic prevention and control, particularly in information sharing, as a bridge between HCPs and patients. Future studies are needed to identify the key actors in and effective approaches for community engagement during pandemics to guide future interventions.

Based on the study findings, we propose several targeted recommendations to enhance the resilience and responsiveness of SRH service delivery during public health emergencies. First, targeted communication strategies should be developed and implemented by healthcare providers to minimize service delays and avoid missed care, thereby reducing adverse health outcomes. Second, it is essential to recognize and address the gender-specific challenges faced by female healthcare providers during pandemics by integrating psychosocial support and workplace protection measures into emergency preparedness plans. Third, telehealth emerged as a key adaptive measure during COVID-19; sustained investment in digital health infrastructure and capacity-building for providers is critical to ensure the continuity of SRH services in future crises. Lastly, the role of communities as trusted intermediaries between clients and providers—particularly in information sharing—should be strengthened through community engagement strategies that build trust, improve provider-patient communication, and facilitate timely access to care. These insights can inform health system strengthening efforts and guide the development of more equitable and resilient SRH service delivery models in future public health emergencies.

### Strengths and limitations

Our study had several strengths. We conducted baseline and endline data collections to track changes and improvements in local SRH services over time. In addition, we collected and analyzed data from both women seeking SRH services and service providers, which yielded a more comprehensive understanding of the challenges faced by the women. Furthermore, we recruited participants from different levels of facilities to ensure the inclusion of diverse perspectives.

Several study limitations should be considered. First, due to the purposive sampling strategy, the findings are specific to the population living in the catchment areas of the public health facilities included in the study. Second, as with all qualitative research, our study draws on a small sample to capture a range of experiences of SRH provision and access; it is not intended to be generalized or quantified. Third, we only focus on the impact of the COVID-19 pandemic on contraception and abortion services, not GBV, IPV and STIs, which were also significantly affected. Fourth, due to the changing epidemic situation in Hubei Province, the endline data for two facilities could not be collected, and some of their data was lost. Fifth, because the quantitative data in this study was only intended to provide a basic set of information about the characteristics of the health facilities included, it is not possible for us to conduct in-depth analysis of the data to compare across the seven different facilities. Moreover, given the different characteristics of the facilities, they cannot be compared horizontally. More importantly, the limited data points restrict our ability to draw any meaningful conclusions of statistical significance.

## Conclusion

The present study highlights the negative impact of the COVID-19 pandemic on SRH services in Wuhan, Beijing, and Changsha, China. This study highlights the lack of health system readiness and the need for comprehensive health system strengthening to ensure the provision of essential sexual and reproductive health services and emergency preparedness for future pandemics. Innovative response strategies, such as telemedicine and community engagement, that can be integrated into routine service delivery are discussed. By incorporating these insights, targeted interventions can be developed to enhance SRH well-being and empower women to navigate their SRH during public health crises.

## Supplementary Information

Supplementary Material 1.

## Data Availability

The data will be available upon request as per the WHO policies. Request for access to data can be sent to alimoa@who.int.
