# Supplementary material for: Evaluating health system barriers to sexual and reproductive health service delivery during the COVID-19 pandemic in China: a mixed-methods study
Source: Reprod Health. 2025 Sep 22;22(Suppl 3):162. doi: 10.1186/s12978-025-02093-z (PMC12455775; doi:10.1186/s12978-025-02093-z)
Supplement: Supplementary file 1 — Supplementary Material 1. [file 12978_2025_2093_MOESM1_ESM.docx]

**Additional file 1**

**Table S1.** Descriptive analysis of health services continuation questionnaire

|  |  | **Baseline survey**  **(n=5)** | **Endline survey**  **(n=5)** |
| --- | --- | --- | --- |
| Has your country defined a national SRH essential health services package (prior to the COVID‐19 pandemic)? | No | 0 | 0 |
|  | Yes | 3 | 1 |
|  | Don’t know | 2 | 4 |
| Has your country identified a core set of essential health services to be maintained during the COVID‐19 pandemic? | No | 0 | 0 |
|  | Yes | 4 | 2 |
|  | Don’t know | 1 | 3 |
| Is there additional government funding allocated to assuring essential health services? | No | 1 | 1 |
|  | Yes | 4 | 1 |
|  | Don’t know | 0 | 3 |
| During the COVID‐19 pandemic, what are the government policies for the following?  Outpatient services | Functional as normal | 1 | 1 |
|  | Limited access | 4 | 1 |
|  | Suspended | 0 | 0 |
|  | Don't know | 0 | 3 |
| Inpatient services | Functional as normal | 0 | 1 |
|  | Limited access | 4 | 1 |
|  | Suspended | 1 | 0 |
|  | Don't know | 0 | 3 |
| Emergency unit services | Functional as normal | 2 | 1 |
|  | Limited access | 2 | 1 |
|  | Suspended | 0 | 0 |
|  | Don't know | 1 | 3 |
| Prehospital emergency care services (e.g., ambulance transport) | Functional as normal | 2 | 2 |
|  | Limited access | 0 | 0 |
|  | Suspended | 0 | 0 |
|  | Don't know | 3 | 3 |
| Community based care | Functional as normal | 1 | 1 |
|  | Limited access | 2 | 1 |
|  | Suspended | 0 | 0 |
|  | Don't know | 2 | 3 |
| Mobile clinics | Functional as normal | 0 | 1 |
|  | Limited access | 0 | 1 |
|  | Suspended | 1 | 0 |
|  | Don't know | 4 | 3 |
| Which of the following services have been disrupted due to COVID‐19? Family Planning and contraception | Completely disrupted | 2 | 0 |
|  | Partially disrupted | 0 | 0 |
|  | Not disrupted | 3 | 2 |
|  | Not applicable | 0 | 3 |
|  | Don't know | 0 | 0 |
| Antenatal care | Completely disrupted | 0 | 0 |
|  | Partially disrupted | 0 | 1 |
|  | Not disrupted | 4 | 1 |
|  | Not applicable | 1 | 3 |
|  | Don't know | 0 | 0 |
| Safe abortion services | Completely disrupted | 1 | 0 |
|  | Partially disrupted | 1 | 1 |
|  | Not disrupted | 3 | 1 |
|  | Not applicable | 0 | 3 |
|  | Don't know | 0 | 0 |
| Post‐abortion care | Completely disrupted | 1 | 0 |
|  | Partially disrupted | 0 | 0 |
|  | Not disrupted | 4 | 2 |
|  | Not applicable | 0 | 3 |
|  | Don't know | 0 | 0 |
| Facility based births | Completely disrupted | 0 | 0 |
|  | Partially disrupted | 0 | 0 |
|  | Not disrupted | 4 | 1 |
|  | Not applicable | 1 | 4 |
|  | Don't know | 0 | 0 |
| Gender based violence | Completely disrupted | 0 | 0 |
|  | Partially disrupted | 0 | 0 |
|  | Not disrupted | 2 | 0 |
|  | Not applicable | 3 | 3 |
|  | Don't know | 0 | 2 |
| Routine immunization services in health facilities | Completely disrupted | 0 | 0 |
|  | Partially disrupted | 0 | 1 |
|  | Not disrupted | 2 | 0 |
|  | Not applicable | 3 | 3 |
|  | Don't know | 0 | 1 |
| Sick child services/IMNCI | Completely disrupted | 2 | 0 |
|  | Partially disrupted | 1 | 1 |
|  | Not disrupted | 2 | 1 |
|  | Not applicable | 0 | 3 |
|  | Don't know | 0 | 0 |
| Outbreak detection and control (for non‐COVID diseases) | Completely disrupted | 0 | 0 |
|  | Partially disrupted | 0 | 1 |
|  | Not disrupted | 5 | 1 |
|  | Not applicable | 0 | 3 |
|  | Don't know | 0 | 0 |
| 24‐hour emergency room/unit services | Completely disrupted | 0 | 0 |
|  | Partially disrupted | 0 | 1 |
|  | Not disrupted | 4 | 1 |
|  | Not applicable | 1 | 3 |
|  | Don't know | 0 | 0 |
| Urgent blood transfusion services | Completely disrupted | 0 | 0 |
|  | Partially disrupted | 0 | 0 |
|  | Not disrupted | 4 | 0 |
|  | Not applicable | 1 | 4 |
|  | Don't know | 0 | 1 |
| Inpatient critical care services | Completely disrupted | 0 | 0 |
|  | Partially disrupted | 0 | 0 |
|  | Not disrupted | 4 | 2 |
|  | Not applicable | 1 | 3 |
|  | Don't know | 0 | 0 |
| Emergency surgery (including obstetric, infection) | Completely disrupted | 0 | 0 |
|  | Partially disrupted | 0 | 1 |
|  | Not disrupted | 4 | 0 |
|  | Not applicable | 1 | 3 |
|  | Don't know | 0 | 1 |
| Other | Completely disrupted | 2 | 0 |
|  | Partially disrupted | 0 | 0 |
|  | Not disrupted | 1 | 0 |
|  | Not applicable | 0 | 3 |
|  | Don't know | 2 | 2 |
| What are the main causes of this disruption(s) and/or change(s) in service utilization?  Closure of outpatient services as per government directive | No | 3 | 2 |
|  | Yes | 2 | 3 |
|  | Don't know | 0 | 0 |
| Closure of outpatient disease specific consultation clinics | No | 1 | 3 |
|  | Yes | 4 | 2 |
|  | Don't know | 0 | 0 |
| Closure of population level cervical cancer screening programs | No | 2 | 3 |
|  | Yes | 2 | 1 |
|  | Don't know | 1 | 1 |
| Decrease in outpatient volume due to patients not presenting | No | 1 | 1 |
|  | Yes | 4 | 4 |
|  | Don't know | 0 | 0 |
| Decrease in inpatient volume due to cancellation of elective care | No | 1 | 1 |
|  | Yes | 4 | 4 |
|  | Don't know | 0 | 0 |
| Inpatient services/hospital beds not available | No | 4 | **4** |
|  | Yes | 0 | 1 |
|  | Don't know | 1 | 0 |
| Insufficient staff to provide services | No | 4 | 2 |
|  | Yes | 1 | 3 |
|  | Don't know | 0 | 0 |
| Related clinical staff deployed to provide COVID‐19 relief | No | 1 | 1 |
|  | Yes | 4 | 4 |
|  | Don't know | 0 | 0 |
| Insufficient Personal Protective Equipment (PPE) available for health care providers to provide services | No | 3 | 5 |
|  | Yes | 2 | 0 |
|  | Don't know | 0 | 0 |
| Unavailability/Stock out of essential medicines, medical diagnostics or other health products at health facilities | No | 5 | 4 |
|  | Yes | 0 | 1 |
|  | Don't know | 0 | 0 |
| Changes in treatment policies for care seeking behavior for fever symptoms (e.g. stay at home policies) | No | 2 | 2 |
|  | Yes | 2 | 3 |
|  | Don't know | 1 | 0 |
| Government or public transport lockdowns hindering access to the health facilities for patients | No | 2 | 4 |
|  | Yes | 3 | 1 |
|  | Don't know | 0 | 0 |
| Financial difficulties during outbreak/lock down | No | 2 | 2 |
|  | Yes | 2 | 2 |
|  | Don't know | 1 | 1 |
| Other (what are the other causes of this disruption and/or changes in service utilization) | No | 5 | 3 |
|  | Yes | 0 | 1 |
|  | Don't know | 0 | 1 |
| What approaches are being used to overcome the disruptions to essential health services in public sector health facilities?  Telemedicine deployment to replace in‐person consults | No | 1 | 1 |
|  | Yes | 4 | 4 |
|  | Don't know | 0 | 0 |
| Task shifting/role delegation | No | 0 | 0 |
|  | Yes | 5 | 5 |
|  | Don't know | 0 | 0 |
| Novel supply chain and/or dispensing approaches for medicines through other channels | No | 2 | 3 |
|  | Yes | 2 | 2 |
|  | Don't know | 1 | 0 |
| Triaging to identify priorities | No | 2 | 3 |
|  | Yes | 2 | 1 |
|  | Don't know | 1 | 1 |
| Redirection of patients to alternate health care facilities | No | 1 | 3 |
|  | Yes | 4 | 2 |
|  | Don't know | 0 | 0 |
| Community outreach to inform on service disruptions and changes | No | 2 | 3 |
|  | Yes | 3 | 2 |
|  | Don't know | 0 | 0 |
| Government removal of user fees | No | 3 | 5 |
|  | Yes | 1 | 0 |
|  | Don't know | 1 | 0 |
| Other (describe what other approaches are being used) | No | 4 | 4 |
|  | Yes | 1 | 0 |
|  | Don't know | 0 | 1 |

**Table S2.** Numbers of participants in the baseline and endline survey

|  | A Grade-A Tertiary Hospital in Beijing | A Grade-A Secondary Hospital in Beijing | A Community Health Center in Beijing | A Grade-A Tertiary Hospital in Wuhan (Changsha for the endline) | A Community Health Center in Wuhan (Changsha for the endline) |
| --- | --- | --- | --- | --- | --- |
| **Baseline survey** | | | | | |
| Health service providers | 4 | 3 | 2 | 3 | 2 |
| Women | 5 | 9 | 9 | 7 | 2 |
| Partners | 1 | 3 | 3 | 1 | 1 |
| Total | 10 | 15 | 14 | 11 | 5 |
| **Endline survey** | | | | | |
| Health service providers | 4 | 3 | 1 | 4 | 3 |
| Women | 3 | 8 | 7 | 7 | 6 |
| Partners | 1 | 1 | 2 | 3 | 2 |
| Total | 8 | 12 | 10 | 14 | 11 |

**Table S3.** Selection of study sites

| Location | Facility | Setting | Assessment |
| --- | --- | --- | --- |
| Beijing | Grade 3A hospital | Urban | Qualitative & Quantitative |
|  | Grade 2A hospital | Urban | Qualitative & Quantitative |
|  | Community health center | Suburban/rural | Qualitative & Quantitative |
| Wuhan | Grade 2A hospital | Urban | Qualitative & Quantitative |
|  | Community health center | Suburban/rural | Qualitative & Quantitative |
| Changsha | Grade 3A hospital | Urban | Qualitative & Quantitative |
|  | Community health center | Urban | Qualitative & Quantitative |
